# Accuracy of a rapid diagnosis test, microscopy and loop-mediated isothermal amplification in the detection of asymptomatic *Plasmodium* infections in Korhogo, Northern Côte d’Ivoire

**DOI:** 10.1186/s12936-022-04133-6

**Published:** 2022-04-02

**Authors:** Edjronké M. A. Benié, Kigbafori D. Silué, Xavier C. Ding, Issa Yeo, J. B. Assamoi, Karim Tuo, Akpa P. Gnagne, Lasme J. C. E. Esso, Jean T. Coulibaly, Serge-Brice Assi, Bassirou Bonfoh, William Yavo, Eliézer K. N’Goran

**Affiliations:** 1 Unité de Formation et de Recherche Biosciences, Laboratoire de Biologie et Santé, Université Félix Houphouët-Boigny, Abidjan 01, 01 BP V34 Côte d’Ivoire; 2 Centre Suisse de Recherches Scientifiques en Côte d’Ivoire, Abidjan 01, 01 BP 1303 Côte d’Ivoire; 3 FIND, Campus Biotech, 9 Ch. des Mines1202, Geneva, Switzerland; 4 Institut Pasteur de Côte d’Ivoire, Abidjan 01, 01 BP 490 Côte d’Ivoire; 5 Centre de Recherche et de Lutte Contre le Paludisme, Institut National de Santé Publique, Abidjan, Côte d’Ivoire; 6 Programme National de Lutte Contre le Paludisme en Côte d’Ivoire, Ministère de la Santé et de l’Hygiène Publique, BP V4, Abidjan, Côte d’Ivoire

**Keywords:** *Plasmodium* spp., Asymptomatic, RDT, Microscopy, LAMP, PCR, Korhogo, Côte d’Ivoire

## Abstract

**Background:**

Highly sensitive and accurate malaria diagnostic tools are essential to identify asymptomatic low parasitaemia infections. This study evaluated the performance of histidine-rich protein 2 (HRP-2) based rapid diagnostic tests (RDTs), microscopy and loop-mediated isothermal amplification (LAMP) for the detection of asymptomatic *Plasmodium* spp. infections in Northern Côte d’Ivoire, using nested polymerase chain reaction (nPCR) as reference.

**Methods:**

A household-based survey was carried out in July 2016, in the health district of Korhogo, involving 1011 adults without malaria symptom nor history of fever during the week before recruitment. The fresh capillary blood samples were collected to detect *Plasmodium* infections using on HRP-2-based RDTs, microscopy and LAMP and stored as dried blood spots (DBS). A subset of the DBS (247/1011, 24.4%) was randomly selected for nPCR analyses. Additionally, venous blood samples, according to LAMP result (45 LAMP positive and 65 LAMP negative) were collected among the included participants to perform the nested PCR used as the reference.

**Results:**

The prevalence of asymptomatic *Plasmodium* spp. infections determined by RDT, microscopy, and LAMP were 4% (95% confidence interval (CI) 2.8–5.3), 5.2% (95% CI 3.9–6.6) and 18.8% (95% CI 16.4–21.2), respectively. Considering PCR on venous blood as reference, performed on 110 samples, the sensibility and specificity were, respectively, 17.8% (95% CI 6.1–29.4) and 100% for RDT, 20.0% (95% CI 7.8–32) and 100% for microscopy, and 93.3% (95% CI 85.7–100) and 95.4% (95% CI 92.2–100) for LAMP.

**Conclusion:**

In Northern Côte d’Ivoire, asymptomatic *Plasmodium* infection was found to be widely distributed as approximately one out of five study participants was found to be *Plasmodium* infected. LAMP appears currently to be the only available diagnostic method that can identify in the field this reservoir of infections and should be the method to consider for potential future active case detection interventions targeting elimination of these infections.

## Background

Diagnostic tools of high accuracy are critical to identify and manage malaria infections, especially in areas aiming for elimination and where the detection of asymptomatic and low-density malaria infections represent a critical challenge [1]. Asymptomatic infections are indeed often sub-patent and fall under the threshold of detection of the usual diagnostic for malaria: microscopy and rapid diagnostic tests (RDT) [2].

Interventions based on active case detection amongst symptomatic and asymptomatic populations can be used to attempt reducing malaria transmission. However, doing so using conventional malaria diagnostics, such as microscopy and RDTs, will miss a significant number of low-density infections and might overestimate the success of such interventions [3, 4]. Hence, if possible, molecular-based detection tools should be integrated into elimination interventions as well as within epidemiological studies [5]. Nucleic acid amplification-based techniques (NAAT) offer markedly improved sensitivity compared to microscopy and RDTs [6]. Such molecular tests are suitable for detection of low density infections, in the range of 5 parasites per microlitre of blood (p/µL) and below, with sophisticated techniques achieving limits of detection in the order of 0.002 p/µL [7]. NAATs are typically not adapted for point-of-care use or for field settings in endemic countries as they often require sophisticated laboratory infrastructure and highly trained laboratory technicians [8]. Yet, loop-mediated isothermal amplification (LAMP) is a simplified NAAT that can be deployed under limited laboratory infrastructure and that is especially amenable to screening activities requiring a high analytical sensitivity. Malaria LAMP assays, that do not require refrigerated storage and that can be interpreted with the naked eye are commercially available and have been shown to provide similar lower limit of detection (LOD) when used in remote settings than polymerase chain reaction (PCR) conducted in reference laboratories [1, 2]. LAMP offers therefore a molecular alternative to microscopy and RDT for active case detection with the potential to catch a significantly higher fraction of all infections than these techniques [8].

When the goal is to interrupt transmission and to eliminate malaria in a given area, there is a need to ideally detect all the infections, including asymptomatic ones, which are usually of low density [6] and only seen by highly sensitive malaria diagnostic tools. In the current study, the diagnostic performance of a selected RDT, microscopy and LAMP were assessed to support the active detection of asymptomatic *Plasmodium* spp. infected individuals in Korhogo, Northern Côte d’Ivoire.

## Methods

### Study area

The study was carried out in the health district of Korhogo, Northern Côte d’Ivoire, from the 17 to the 28 July 2016. The population of Korhogo was estimated in 2014 at 286′071 inhabitants [9]. The climate is characterized by two seasons: a rainy season that occurs from May to October and a dry season from November until April. The yearly rainfall is 1200 mm and the average temperature is 26.6 °C [10]. Malaria National strategy targeted control is this area. Korhogo showed over the decade 2004–2013 seasonal distribution of malaria cases. The highest numbers of cases occurred starting from June to November and the lowest malaria counts appeared from December to April [11]. In 2013, prevalence of clinical malaria in this area was 14.7% [12]. For the supply of water for drinking, irrigation, agricultural activities and market gardening, a dam was built in 1972. *Anopheles gambiae* is the most prevalent vector and *Plasmodium falciparum* the predominant species of malaria parasites, in this district [10, 13].

### Study design and procedures

The study is a cross-sectional household survey. As described in a recent study carried out in Korhogo [13], the households were randomly selected from 30 demographic zones. Activities were conducted over 10 days by three teams, each including a medical doctor or a nurse, a laboratory technician, and questionnaire administrator. Adult participants (≥ 18 years old) were enrolled using a household-based approach. Inclusion criteria were: (i) absence of fever (axillary temperature > 37.5 °C) or self-reporting history of fever during the last seven days; (ii) self-reporting of no more than one malaria symptoms, such as headache, shiver, sweat, nausea, vomiting, giddiness, tiredness, abdominal pain; (iii) no self-reporting of anti-malarial treatment over the four previous weeks; (iv) written informed consent of the participant.

A finger-prick blood sample was collected for *Plasmodium* spp. infection detection using routine RDT for case management (One Step Malaria Antigen *P. falciparum* (HRP2), FIRST RESPONSE®), microscopy, and LAMP (Loopamp™ MALARIA Pan/Pf Detection Kit, Eiken Chemical Company). Capillary blood samples were also collected and stored on filter paper (Whatman 903). The dried blood spot (DBS) was used for subsequent nested PCR analysis.

At the end of each day, samples collected for LAMP and immediately mixed in extraction buffer (see below) were transferred to the laboratory of the *Centre Hospitalier Régional* (CHR) of Korhogo health district, where the assay was performed. A subset of the participants, positive and negative based on LAMP results were invited to go to CHR following day, where a 15 mL venous blood sample was collected and stored at 4 °C to support specimen banking activities. Within 24 h, these samples were aliquoted and frozen at − 20 °C in the CHR laboratory in Korhogo and transferred within the following 7 days at Abidjan where there were stored at − 80 °C for the subsequent PCR analyses.

### Ethical consideration

The study was approved in 2016 by the National Ethic and Research Committee of Côte d’Ivoire (NCER) (N/Réf: 120/MSHP/CNER-dk), National Malaria Control Programme (NMCP) and the local health district authorities. In order to comply with the NMCP recommendations pertaining to the national policy on the management of malaria cases, asymptomatic participants positive to *Plasmodium* spp. were not treated. All of them received an insecticide-treated bed net (ITN). They were also advised, if any malaria symptom would appear, to promptly go to a health centre for malaria diagnosis and, if needed, treatment.

### Laboratory procedures

#### RDT

RDT was done using a fresh capillary blood. RDT (RDT, One Step Malaria Antigen *P. falciparum* (HRP2), FIRST RESPONSE®) used routinely in Côte d’Ivoire and detecting only *P. falciparum*, was performed according to the manufacturer’s instructions. Results were obtained after 15 min.

#### Microscopy

Microscopy was performed using fresh capillary blood. A thick and thin blood film was made on a slide and air dried on site. Thin blood film was fixed in methanol. Both thick and thin blood film were stained with Giemsa 10% for 20 min. Slides were transferred to the *Centre Suisse de Recherches Scientifiques en Côte d’Ivoire* (CSRS) in Abidjan and read under oil immersion with × 100 objective by two experienced technicians who were blinded to other diagnostic results. For quality control, 10% of the smears were read two times by the technicians. In case of discrepancies, a third reader was involved to resolve it. The thin blood films were used to identify the species of malaria parasites. The parasitaemia was estimated in the thick blood films by the number of parasites per 200 white blood cells (WBCs) calculated by assuming 8000 WBC/µL. A slide was classified as negative if no *Plasmodium* asexual forms or gametocytes was found after counting 500 WBCs.

#### LAMP

The LAMP reactions were conducted according to the manufacturer’s recommendations using a standard heat block and results were read under a UV lamp. Sixty microlitres of fresh capillary blood were collected from finger prick and mixed immediately in an equal volume of extraction buffer (5 M NaCl, 100 mM Tris HCL pH 6.4, 10% SDS). The mixture was stored at ambient temperature for no more than 24 h before processing using a “boil and spin” method described elsewhere [14]. Extracted DNA samples were first tested using a Pan LAMP assay detecting all human-infecting of *Plasmodium* spp. Then, all the positive samples were further tested using a *P. falciparum* specific LAMP assay (Pf LAMP). The samples were classified as Malaria negative (Pan LAMP negative), *P. falciparum* positive (Pan and Pf LAMP positive) or non-falciparum positive (Pan LAMP positive and Pf LAMP negative). Results were available within one hour, by fluorescence’s visualization under UV light.

#### Dried blood spot

The dried blood spots were collected onto filter papers (Whatman 903), from fresh capillary blood. They were air-dried and packaged in individual sealable bags containing a desiccant. They were initially stored at CSRS during six months at room temperature before being transferred at − 80 °C for long-term storage until the DNA extraction could be performed three years later, at the *Centre de Recherches et de Lutte contre le Paludisme* (CRLP) of the *Institut National de Santé Publique de Côte d’Ivoire* (INSP). Before this long-term storage (3 years), all PCR analyses had failed. Problems were solved at the end of this period, after prospecting procedure, reagent, and primers. There is no data about temperature monitoring during long-term storage.

#### Venous blood

The venous blood was collected in anticoagulant tubes containing ethylenediaminetetraacetic acid (EDTA). One aliquot of 200 µL were retained for PCR analyses and stored as described above. The DNA extraction could be made four years later, at CRLP.

#### DNA extraction

The DNA was extracted from capillary blood samples stored as DBS and from liquid venous blood samples using Quick-DNA™ Miniprep Kit and Quick-DNA™ Miniprep Plus Kit (Zymo research corp., USA), respectively, according to the manufacturer’s instructions. DNA from DBS and venous blood were eluted with 100 µL and 50 µL of DNA elution buffer, respectively. The extracted DNA was stored at − 20 °C until the PCR analyses could be completed.

#### PCR analyses

PCR analyses were conducted using a nested PCR protocol as described elsewhere [15]. This method allows to identify and discriminate between *P. falciparum, Plasmodium malariae*, *Plasmodium ovale* and *Plasmodium vivax* species. PCR was repeated in triplicate for all samples showing discrepancy between PCR and LAMP results and for an equal number of randomly selected concordant samples. PCR was considered positive if at least one the repeat was positive and negative if all three did not show any DNA amplification.

#### Data analyses

The statistical analysis was conducted using STATA 12 (Stata Corp, Texas, USA). The Prevalence of malaria infections amongst asymptomatic study participants was determined by RDT, microscopy and LAMP in overall screening population with interval confidence at 95%. The diagnostic accuracy estimates were established using nested PCR as the reference, elaborated on the sample types. Sensitivity, specificity, positive predictive value (PPV) and negative predictive value (NPV) of RDT, microscopy and LAMP were calculated. Kappa value (k) was determined to assess the agreement between diagnosis approaches. Kappa result was interpreted as follows: values ≤ 0 as indicating no agreement and 0.01–0.20 as none to slight, 0.21–0.40 as fair, 0.41– 0.60 as moderate, 0.61–0.80 as substantial, and 0.81–1.00 as almost perfect agreement [16].

## Results

### Study populations characteristics

**Fig. 1:**
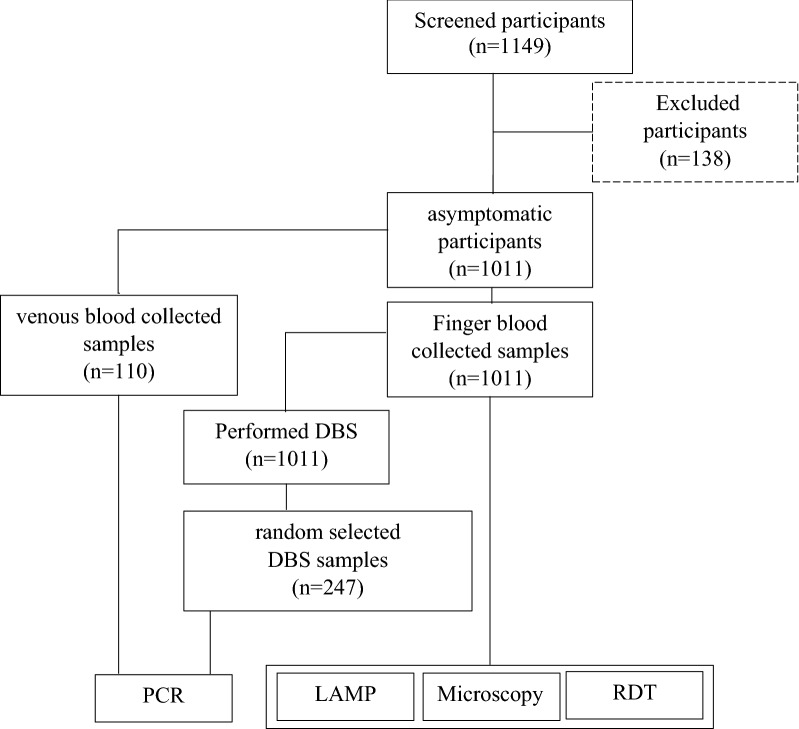
Flow chart of study

One thousand and eleven (1011) asymptomatic participants were enrolled in the current study. The mean age was 35.1 years old (range 18–87), and female represented 69% of this population. They have all been subjected to the malaria RDT, microscopy and LAMP tests. DBS were collected for all of them but only 247 randomly selected samples were used for the PCR analyses. Molecular analyses were also performed for 110 venous blood samples collected (representing 45 LAMP positive and 65 LAMP negative) (Fig. 1).

### Prevalence of *Plasmodium* asymptomatic carriers

**Fig. 2:**
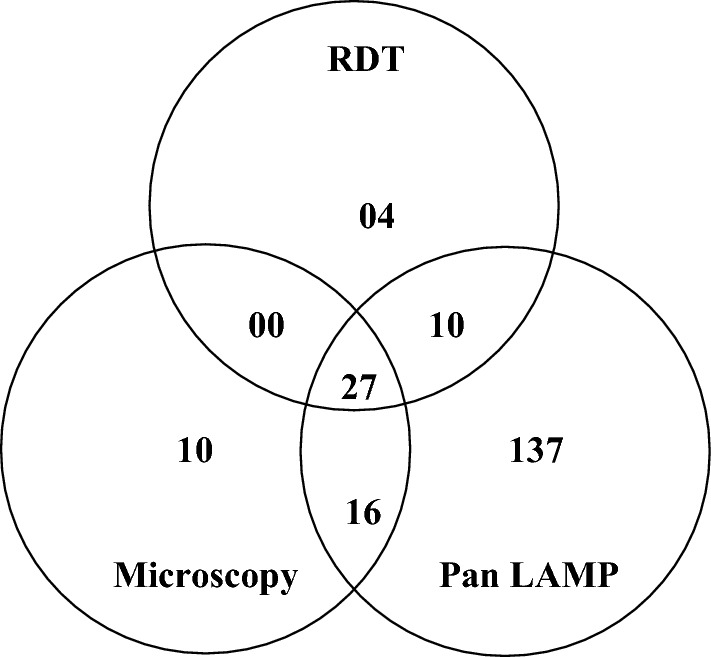
Venn diagram showing the distribution of positive RDT, microscopy and Pan LAMP results

Out of the 1011 asymptomatic participants included, the RDT, microscopy and Pan LAMP detected respectively 41, 53 and 190 asymptomatic *Plasmodium* carriers, resulting in prevalence of 4% (95% CI 2.8–5.3), 5.2% (95% CI 3.9–6.6) and 18.8% (95% CI 16.4–21.2), respectively. *P. falciparum* was the only species detected by microscopy. Of the 190 Pan LAMP positive samples, 82.1% (156/190) were found to be *P. falciparum* (positive by Pf LAMP, including potential mixed-species infections) and 17.9% (34/190) to be non-falciparum (negative to Pf LAMP) (Table 1). Of all LAMP positives, 19.5% (37/190) were detected by RDT, 22.6% (43/190) by microscopy; and 72.1% (137/190) were missed by both (RDT and microscopy) (Fig. 2).

**Table 1:** Prevalence of *Plasmodium* spp.

Diagnostic approach	N	*Plasmodium* spp.		*Plasmodium falciparum*		Non-falciparum	
		No. of infected	% (95% CI)	No. of infected	% (95% CI)	No. of infected	% (95% CI)
HRP-2-RDTs	1011	41	4.0 (2.8–5.3)	41	100	ND	ND
Microscopy	1011	53	5.2 (3.9–6.6)	53	100	0	0
LAMP	1011	190	18.8 (16.4–21.2)	156	82.1 (76.6–87.6)	34	17.9 (12.4–23.4)

*HRP-2-RDTs* histidine-rich protein 2 based rapid diagnostic tests, *LAMP* loop-mediated isothermal amplification, *N* number of samples examined, *No* number*, CI* confidence interval, *ND* not determined

### Performance of malaria diagnosis tool with PCR on venous blood as reference

Nested PCR was conducted on 110 venous blood samples collected 24 h after the initial capillary blood sample used for the other diagnostic methods. Individuals who provided a venous sample were selected based on their LAMP results. Discrepancy result between LAMP and PCR represents 5.4% (6/110) all samples. Previously, PCR was repeated for 8 discrepant samples, in addition 8 randomly selected concordant samples were used as control (4 LAMP negative-PCR negative and 4 LAMP positive-PCR positive). Repeated PCR samples controls results were same as initial results (Table 2).

**Table 2:** Discrepancies results between LAMP and PCR

DNA template to PCR	N	Before PCR triplicate					After PCR triplicate				
		Discrepancies	LAMP + PCR-	LAMP − PCR +	LAMP − PCR −	LAMP + PCR +	Discrepancies	LAMP + PCR −	LAMP − PCR +	LAMP − PCR −	LAMP + PCR +
		n (%)	n	n			n (%)	n	n		
Venous blood	110	8 (7.3)	3	5	60	42	6 (5.4)	3	3	62	42
DBS	247	51 (20.6)	51	0	183	13	50 (20.2)	50	0	183	14

*DNA* deoxyribonucleic acid, *DBS* dried blood spot, *N* number of samples examined, *LAMP* loop mediated-isothermal amplification, *PCR* polymerase chain reaction, +  positive, − negative

Of the 45 samples positive by LAMP for *Plasmodium* spp., 93.3% (42/45) were positive by PCR; and of the 65 LAMP-negative samples, 4.6% (3/65) were found to be positive by PCR. Of the 110 venous blood samples tested, PCR found 45 to be positive for *Plasmodium* spp. Species identification showed that 93.3% (42/45) were *P. falciparum* (mono and mixed-species infection) and 6.7% (3/45) were non-falciparum. Details of *P. falciparum* infection revealed that 40% (18/45) samples were mono infection and 53.3% (24/45) were mixed-species infections: 18 *P. falciparum* and *P. ovale*; 2 *P. falciparum* and *P. malariae*; 4 *P. falciparum* and *P. ovale* and *P. malariae*. Amongst the 3 non-falciparum, 1 mono infection to *P. malariae* and 2 mixed-species infections to *P. ovale* and *P. malariae* were found (Table 3).

**Table 3:** *Plasmodium* species repartition according to venous blood PCR selected from 45 LAMP positive 65 negative

Malaria diagnosis tools tested	Nested PCR							
		Genus	Species					
	N	Positive	Pf	Pm	Pf-Po	Pf-Pm	Po-Pm	Pf-Po-Pm
		n	n	n	n	n	n	n
HRP-2-RDT								
Negative	102	37	12	01	17	02	02	03
Positive	08	08	06	00	01	00	00	01
Microscopy								
Negative	101	36	13	01	16	01	02	03
Positive	09	09	05	00	02	01	00	01
Pan LAMP								
Negative	65	03	03	00	00	00	00	00
Positive	45	42	15	01	18	02	02	04
Pf LAMP								
Negative	05	04	01	01	00	00	02	00
Positive	40	38	14	00	18	02	00	04
Nested PCR	110	45	18	01	18	02	02	04

*HRP-2-RDTs* histidine-rich protein 2 based rapid diagnostic tests, *LAMP* loop mediated-isothermal amplification,* N* number of samples examined,* n* number*, Pf Plasmodium falciparum*,* Pm Plasmodium malariae*, *Po Plasmodium ovale*

The kappa agreement was found to be slight between RDT and PCR (κ = 0.20), fair between microscopy and PCR (κ = 0.22), almost perfect between Pan LAMP and PCR (κ = 0.88), almost perfect between Pf LAMP and PCR (κ = 0.84) (Table 4).

**Table 4:** Diagnosis accuracy compared to nested PCR based on venous blood and DBS as DNA template

DNA template to PCR	Sensitivity (95% CI)	Specificity (95% CI)	PPV (95% CI)	NPV (95% CI)	Agreement % (Kappa)
Venous blood, N = 110					
HRP-2-RDT	17.8 (6.1–29.4)	100	NA	NA	66.4 (0.20)
Microscopy	20.0 (7.8–32.1)	100	NA	NA	67.3 (0.22)
Pan-LAMP	93.3 (85.7–100)	95.4 (90.1–100)	NA	NA	94.5 (0.88)
Pf-LAMP	97.4 (92.2–100)	100	NA	NA	97.6 (0.84)
DBS, N = 247					
HRP-2-RDT	35.7 (07–64.2)	97.8 (96–99.7)	50 (12.3–87.7)	96.2 (93.7–98.6)	94.3 (0.39)
Microscopy	57.1 (27.5–86.8)	97 (94.8–99.2)	53.3 (24.7–81.9)	97.4 (95.3–99.4)	94.7 (0.52)
Pan LAMP	100	78.5 (73.2–83.8)	21.9 (11.5–32.2)	100	79.8 (0.29)
Pf LAMP	ND	ND	ND	ND	ND

*DNA* deoxyribonucleic acid, *DBS* dried blood spot, *HRP-2-RDTs* histidine-rich protein 2 based rapid diagnostic tests, *Pf Plasmodium falciparum*, *LAMP* loop-mediated isothermal amplification, *CI* confidence interval, *PPV* positive predictive value, *NPV* negative predictive value, *NA* not applicable, *ND* not determined

After matching DBS and venous blood samples, 43 individuals were tested using both sample types. All of the 29 venous blood samples negative by PCR were also found to be negative when tested by DBS. Of the 14 venous blood samples positive by PCR, only 3 (21.4%, 3/14), were found to be positive when tested by DBS. This result suggests that PCR performed in this study, is less sensitive when using DBS than liquid venous blood, that the integrity of the DBS has been affected, or a combination of both (Table 5).

**Table 5:** PCR result after overlapping DBS and venous blood samples

BDS-PCR	Venous blood-PCR		p value
	Positive, N = 14	Negative, N = 29	
Positive, n/N (%)	3/14 (21.4)	0/29 (0%)	
Negative, n/N (%)	11/14 (78.6)	29/29 (100%)	0.029

*DBS* dried blood spot, *PCR* polymerase chain reaction,* N* number of samples examined,* n* number

### Weakness of DBS-based data

PCR was performed using capillary blood stored on DBS for 247 out 1011 samples (24.4%). Discrepancy result between LAMP and DBS-nPCR represented 20.2% (50/247) (Table 2).

The diagnostic accuracy estimates of RDT, microscopy and LAMP together with kappa agreement between these methods and nested PCR results are presented in Table 4. The kappa agreement was found to be fair between RDT and DBS-nPCR (κ = 0.39), moderate between microscopy and DBS-nPCR (κ = 0.52) and fair between LAMP and PCR (κ = 0.29).

## Discussion

Asymptomatic carriers of *Plasmodium* spp. parasites are important reservoirs that can significantly contribute to malaria transmission in endemic areas. Such infections come with specific challenges: they require to be actively detected, since carries will not be presenting themselves to health centres, and many of them have a parasitaemia too low to be reliably detected by routine malaria diagnosis tools. Hence, more sensitive malaria diagnostic tools that are compatible with active case detection interventions are required.

In this study, the performance of RDT, microscopy and LAMP were evaluated in such a context to detect active asymptomatic *Plasmodium* spp. infections in Korhogo, Northern Côte d’Ivoire.

Malaria cases in Korhogo, over the decade 2004–2013, occurred at low levels during dry seasons, and high levels during rainy seasons, with highest number of cases typically seen in July [11]. These data were tightened up by a cross-sectional survey, carried in July 2014 (rainy season) and March 2015 (dry season), which concluded that, asymptomatic malaria infection was significantly associated with season, with higher risk of asymptomatic malaria carriage during the rainy season [13]. Data reported here were collected in July, a high malaria transmission period, in line with the high proportion of asymptomatic *Plasmodium* spp. carriage found.

The finding of non*-*falciparum infections was not negligeable, as it was found in 6.7% to 17.9% of the asymptomatic *Plasmodium* spp. carriers. PCR revealed it was primarily *P. malariae* and *P. ovale*. This is in line with previously reported national pattern, showing that 94.5% of the detected *Plasmodium* infections were due to *P. falciparum*, whilst *P. malariae* and *P. ovale* accounted for 5.1 and 0.4%, respectively [17]. RDT, used in this study, targets *P. falciparum* only, so non-falciparum infections were missed.

RDT and microscopy, which typically have a limit of detection in the range of 50 to 200 p/µL [18], showed a prevalence of asymptomatic *P. falciparum* infections in the range of 4% to 5%. LAMP revealed approximately fourfold more infections, with 18.8% of all tested sample being positive. This shows that not only asymptomatic carriage is significantly present in the study area but also illustrate that routine diagnostic methods miss approximately three quarters of all asymptomatic infections that can be detected using more sensitive DNA-amplification method, such as LAMP which can deliver a LOD in the range of 1 to 5 p/µL of blood [19, 20]. Similar LAMP results for asymptomatic infection detection have been reported in other studies [2, 21–25]. LAMP allowed here to screen a relatively high number of individuals, more than a thousand, over a short period of time, with more than 100 samples tested per day in average over 10 days, showing that this method is compatible with large-scale high-throughput screening campaigns. Furthermore, laboratory technicians received 3 days of training, including theory and practical; and result was available within one hour.

In this study, diagnostic accuracy was assessed using PCR, with DNA template from DBS and venous blood. A process from DBS showed that 20% discrepancy remained between LAMP and PCR despite performing triplicate PCR on initially discrepant samples. Diagnostic accuracy estimates using PCR as reference show that LAMP has a high sensitivity and NPV but a low specificity and PPV. Kappa statistic showed only a “fair” agreement between LAMP and PCR. It cannot be excluded here that, while PCR was performed in a central reference laboratory, the long-term storage of the samples before their analyses by PCR might have prevent PCR to detect samples with an initially low parasitaemia and in which DNA might have degraded during storage [26]. This hypothesis would explain the high sensitivity but low specificity of LAMP when compared to PCR. Moreover, in this study, the volume of buffer used to eluate DNA from DBS was twice that of venous blood samples. Discrepancy between LAMP and PCR on venous blood DNA template was lower (5%). Compared to the routine malaria diagnosis tools, LAMP accuracy was higher and its agreement with PCR was almost perfect, in agreement with a previous study [27]. One study reported that the sensitivity of PCR for malaria on DNA extracted from DBS is approximately half of the sensitivity of PCR on DNA extracted from the whole blood [28].

Despite this high accuracy of LAMP, discrepant result remained, as reported elsewhere [1, 25, 29]*.* These discrepancies (LAMP positive-PCR negative or LAMP negative-PCR positive) is often observed. In some studies, the reason of the discrepancy was unexplained [23, 25]. In some others, it is explained by the subjectivity of LAMP result as they are based on turbidimetry or fluorescence measures. A study conducted in northern Peruvian Amazon, tried to explain LAMP negative-PCR positive, by a possible inhibitory effect of high DNA concentrations on the LAMP reaction. This last study reported that LAMP detection failures occurred whatever high parasite densities [22]. Another hypothesis to explain such discrepancies is that of stochastic effects when comparing two methodologies which have very close limits of detection, which is typically the case when comparing reference standardized PCR and LAMP assays. In this study context, stochastic effects with samples around the detection threshold, may apply to PCR based on venous blood, but not to DBS. The fact that repeats of the PCR yielded, in some cases (02/08, 25% changed vs 06/08, 75% unchanged results), a different result than the one observed initially seem to substantiate this hypothesis when samples with parasite densities on the border of the detection are tested, resulting in a low reproducibility [19].

A limitation of this study was the unavailable of parasites quantification by qPCR in other to detect the LAMP threshold in the field setting. Given that the venous blood samples were selected (45 LAMP positive of 110 tested), PPV and NPV are not appropriate metrics as these are affected by the overall prevalence, which is artificially selected for these samples.

## Conclusion

This study showed the high accuracy of LAMP to detect active asymptomatic *Plasmodium* carrier in the field settings. As compared to DBS, venous blood was the best DNA template for high PCR quality. In Northern Côte d’Ivoire, asymptomatic *Plasmodium* infection was widely distributed. Hence, to move toward elimination in such malaria settings, the use of highly sensitive method that are amenable to point-of-care or quasi point-of-care implementation should be considered as a potential tool to enable effective mass test and treat campaigns.

## Data Availability

The datasets supporting the conclusions of this article are included within the article. Raw data used for analysis of the study are available from the corresponding author on reasonable request.

## Abbreviations

CHR: Centre Hospitalier Régional
CI: Confidence interval
CRLP: Centre de Recherches et de Lutte contre le Paludisme
CSRS: Centre Suisse de Recherches Scientifiques en Côte d’Ivoire
DBS: Dried blood spot
DNA: Deoxyribonucleic acid
HRP-2: Histidine-rich protein 2
RDT: Rapid diagnostic test
INSP: Institut National de Santé Publique de Côte d’Ivoire
LAMP: Loop-mediated isothermal amplification
LOD: Lower limit of detection
NAAT: Nucleic acid amplification-based techniques
NCER: National Ethic and Research Committee in Côte d’Ivoire
NMCP: National Malaria Control Programme
NPV: Negative predictive value
PCR: Polymerase chain reaction
PPV: Positive predictive value
